# An annotated cucumber leaf image dataset for detection of visible damage and abnormal color symptoms in complex cultivation environments

**DOI:** 10.3389/fpls.2026.1962647

**Published:** 2026-10-02

**Authors:** Batyrkhan Omarov, Sayat Ibrayev, Nurbibi Imanbayeva

**Affiliations:** 1 Joldasbekov Institute of Mechanics and Engineering, Almaty, Kazakhstan; 2 International Information Technology University, Almaty, Kazakhstan; 3 Narxoz University, Almaty, Kazakhstan; 4 Satbayev University, Almaty, Kazakhstan

**Keywords:** bounding-box annotation, cucumber leaf abnormality, image dataset, object detection, precision agriculture, visible damage, visible leaf phenotype

## Abstract

Field-deployable plant-health vision systems require datasets that preserve natural variation in illumination, scale, occlusion, and background clutter. This Data Report describes a fixed public release of 500 RGB JPEG images of cucumber leaves collected at three protected-cultivation facilities in Kazakhstan. The collection contains 250 healthy-leaf images and 250 affected-leaf images. Decoder-level inspection confirmed three native image dimensions: 3840 × 2160 pixels (387 images), 4608 × 3456 (112), and 4000 × 2252 (one). The affected subset is paired with 250 text files containing 2,705 rectangular annotations in normalized YOLO center-coordinate format: 1,741 class-0 boxes identify visible non-green color and 964 class-1 boxes identify visible tissue damage. Class 1 includes visible tissue deterioration regardless of whether the apparent cause is disease-associated, mechanical, pest-related, senescent, or otherwise uncertain. The two phenotype labels are operationally non-exclusive: 215 affected images contain both classes, and 116 contain at least one positive-area cross-class box overlap. These labels describe observable phenotypes and do not provide pathogen, disease-species, cultivar, or causal diagnoses. The release supplies bounding boxes, not pixel-level masks; it therefore supports image classification, object detection, and rectangular region localization, but not conventional supervised segmentation. A complete decoder, structure, coordinate, exact-hash, filename, and coarse perceptual-hash audit confirmed one-to-one affected image-label pairing and valid five-field rows, while identifying 42 boundary-crossing boxes in 36 files, 19 byte-identical pairs, and three additional high-similarity candidate pairs. The intentionally balanced final folders are a curated sample and must not be interpreted as natural prevalence. The dataset is publicly available in Mendeley Data, version 1, under CC BY 4.0 at https://doi.org/10.17632/mb3vnhpk8z.1.

## 1 Introduction

Cucumber leaves may exhibit visible changes caused by pathogens, pests, senescence, nutrient imbalance, environmental stress, mechanical injury, or imaging conditions. Common appearances include yellowing, browning, spots, necrotic tissue, deformation, and loss of leaf integrity. Image-based methods offer a non-destructive means of monitoring these appearances, and convolutional models have demonstrated the general feasibility of recognizing plant-health symptoms from RGB images (Mohanty et al., 2016; Ferentinos, 2018). However, an image alone cannot establish etiology, and a visually abnormal region should not be presented as a pathogen-confirmed disease diagnosis without independent biological evidence.

Dataset composition strongly influences the validity of computer-vision evaluation. Controlled-background images can simplify boundaries and illumination, whereas cultivation scenes include overlapping leaves, stems, supports, soil or greenhouse materials, highlights, shadows, and substantial scale variation. PlantDoc and FieldPlant were developed to improve realism and spatial context in field imagery (Singh et al., 2020; Moupojou et al., 2023), while cross-domain analyses have shown that laboratory-to-field shift remains a major limitation (Wu et al., 2023). Dataset-centered reviews therefore emphasize transparent label semantics, challenge diversity, integrity checks, and reproducible partitioning (Xu et al., 2024). Cucumber-specific resources vary in task, scale, geography, and annotation granularity (Zhang et al., 2020, 2021; Khan et al., 2022; Omer et al., 2022; Sultana et al., 2023; Wang and Liu, 2024). **Table 1** makes these differences explicit and records ‘NR’ when the cited publication does not report a requested attribute; this avoids inferring unsupported dataset properties.

**Table 1:** Comparison of cited cucumber image resources with the present fixed release.

Study or resource	Original images; classes	Annotation supplied	Native resolution	Geographic/acquisition context	Complementarity or limitation
Zhang et al. (2020)	NR; 4 image classes	Image-level labels: healthy, powdery mildew, downy mildew, and combined infection	NR	Natural complex greenhouse; collection site NR	Complex-background classification; no released spatial labels reported
Zhang et al. (2021)	600; 3 disease classes (200 each)	Image-level labels	1024 × 682 after standardization	Anhui, China; agricultural-IoT image repository	Small-sample classification without released phenotype boxes
Khan et al. (2022)	600–900 original; 6 classes; 12,000 after augmentation	Image-level labels	NR	Public scan dataset; acquisition origin NR	Augmented classification set; augmented images are not independent acquisitions
Omer et al. (2022)	4,869 reported in Methods; 6 classes	Image-level labels	NR	Farms and natural scenes, Kurdistan Region of Iraq	Causal disease/pest categories rather than phenotype localization
Sultana et al. (2023)	1,280 original; 8 classes; 6,400 after augmentation	Image-level labels	512 × 512	Field acquisition, Bangladesh	Expert-supported classification; no released boxes reported
Wang and Liu (2024)	4,740; 5 image classes; 14,916 objects	Pascal VOC bounding boxes	Native NR; 224 × 224 model input	Greenhouse, Shouguang, Shandong, China	Disease-category localization at larger scale
Present release	500; 250 healthy and 250 affected; 2 box classes	2,705 YOLO boxes on affected images; healthy negative scenes	3840 × 2160 modal; 3840 × 2160 min; 4608 × 3456 max	Three protected-cultivation facilities, Kazakhstan	Phenotype-only color/damage boxes; fixed-release audit and explicit reuse constraints

This Data Report characterizes a compact cucumber leaf image resource collected in complex cultivation environments. The manuscript title has been restricted to visible damage and abnormal color symptoms because the repository does not contain pathogen-confirmed diagnoses. Likewise, segmentation has been removed from the title because version 1 provides rectangular boxes rather than pixel masks. The purpose is to document how the images were gathered and filtered, explain annotation meaning and file organization, report descriptive and integrity analyses of the fixed release, identify limitations, and provide defensible guidance for reuse. No model-comparison experiment or study-specific performance result is reported.

## 2 Materials and methods

### 2.1 Dataset identity and public repository

The fixed dataset is publicly deposited in Mendeley Data under the repository title “An Annotated Image Dataset for Cucumber Leaf Disease Detection and Segmentation in Complex Orchard Environments,” version 1 (Omarov et al., 2026). It was published on 3 August 2026 with DOI 10.17632/mb3vnhpk8z.1 and a CC BY 4.0 licence. That repository title is the formal citation identifier and is deliberately distinguished from the manuscript title, “An Annotated Cucumber Leaf Image Dataset for Detection of Visible Damage and Abnormal Color Symptoms in Complex Cultivation Environments.” The legacy repository words orchard, disease detection, and segmentation do not describe the crop system, diagnostic evidence, or annotation type in version 1: the images come from protected-cultivation facilities, the labels are phenotype-only, and the annotations are rectangles rather than masks. Because version 1 is immutable, these metadata terms are not silently rewritten here. A future metadata-only version 1.1 should retain the version-1 citation, add an explicit terminology note and changelog, and use cultivation, affected leaves, and bounding boxes in user-facing metadata.

Images were collected at the BRB APK greenhouse complex in Almaty and protected-cultivation facilities in Shymkent and Kyzylorda, Kazakhstan. Filename parsing classified 113/500 files (22.6%) as date-bearing and 387/500 (77.4%) as sequence-derived. Of the dated files, 112 encode 20 March 2023 and one encodes 15 December 2025. Filename-derived dates and frame families support coarse grouping, but they are not substitutes for a verified per-image acquisition manifest. Dataset assembly and annotation export were completed in 2026. Version 1 is treated as immutable; corrected coordinates, duplicate removal, metadata enrichment, or new annotations should be deposited as a separately numbered version with a changelog.

### 2.2 Image acquisition, eligibility filters, and metadata limitations

The fixed release contains full-resolution RGB JPEGs acquired with smartphone-class cameras. Decoder-level inspection found 387 images at 3840 × 2160 pixels, 112 at 4608 × 3456, and one at 4000 × 2252; the minimum, maximum, and modal dimensions are therefore 3840 × 2160, 4608 × 3456, and 3840 × 2160, respectively. Images were taken at different viewpoints and distances while retaining surrounding foliage and cultivation infrastructure. The 500 released images were manually selected after acquisition. Screening and condition grouping were not blinded: selectors could see the whole image and knew the provisional healthy/affected grouping. The initial candidate-pool size and rejection counts by reason were not recorded, so no attrition diagram or natural prevalence estimate can be reconstructed. The exact release-eligibility decision rules are operationalized in **Supplementary Table S1**.

Quality screening used a whole-image visual decision sequence. A file was excluded if it could not be decoded, no cucumber leaf was identifiable, blur prevented phenotype interpretation, over- or underexposure made the visible leaf region uninterpretable, or the frame was an obvious duplicate during initial selection. Mild softness, clutter, shadow, highlight, overlap, and partial occlusion were retained when the visible leaf condition remained interpretable. A released image was placed in the healthy folder only when visual inspection found no clear non-green color departure, necrotic or missing tissue, tearing, collapse, or other obvious abnormality on the visible cucumber leaves. This was visual judgment rather than microscopy, pathology testing, or latent-disease exclusion; subtle, early, hidden, or etiologically ambiguous abnormalities may therefore remain in the healthy subset. Images with a clear visible abnormality were assigned to the affected subset, and uninterpretable images were excluded rather than forced into either group.

Version 1 does not contain a per-image site manifest, per-location contribution totals, plant identifiers, total number of sampled plants, cultivar names, exact camera makes or models, complete EXIF records, standardized illumination measurements, or a recorded screening log. These details cannot be reconstructed reliably and are not inferred in this report. Their absence limits site-stratified, cultivar-specific, plant-independent, device-transfer, and attrition analyses. Future versions should include one metadata row per image with site, acquisition session, plant, cultivar, device, date-time, distance, illumination descriptors, screening outcome, and rejection reason. The release-level data dictionary and the unavailable fields are enumerated in **Supplementary Table S2**.

### 2.3 Rectangle annotation protocol and quality control

The 250 affected-leaf images were annotated in Label Studio using rectangular regions. Annotators inspected each image and drew a separate rectangle around each visible region that met either class definition. Multiple boxes can occur in one image. Class 0 (color) denotes a visible departure from normal green appearance, including yellow or brown regions, without requiring visible tissue loss. Class 1 (damage) denotes visibly deteriorated, necrotic, torn, missing, eroded, or collapsed tissue; it includes non-disease-associated damage when the apparent cause could be mechanical injury, pest feeding, senescence, environmental stress, or another uncertain process. Neither class is a pathogen label. The classes are not operationally exclusive and no precedence rule was imposed. When one region met both definitions, annotators could draw a color box and a damage box over the same or partly shared area rather than forcing a single causal category. Across the release, 215/250 affected images (86.0%) contain both classes, 24 (9.6%) contain color only, and 11 (4.4%) contain damage only. Using positive-area overlap defined as IoU > 10^-6^, 116/250 affected images (46.4%) contain at least one overlapping cross-class pair, totaling 255 pairs; median IoU is 0.0159, maximum IoU is 0.3391, 32 pairs have IoU ≥ 0.10, and none has IoU ≥ 0.50. Users should therefore treat the targets as coexisting visual phenotypes, not mutually exclusive disease species. Regions that could not be placed within either visible definition were not assigned a causal label. **Figure 1** illustrates the interface, and **Supplementary Figure S1** shows representative healthy, affected, and boundary-audit examples.

**Figure 1:**
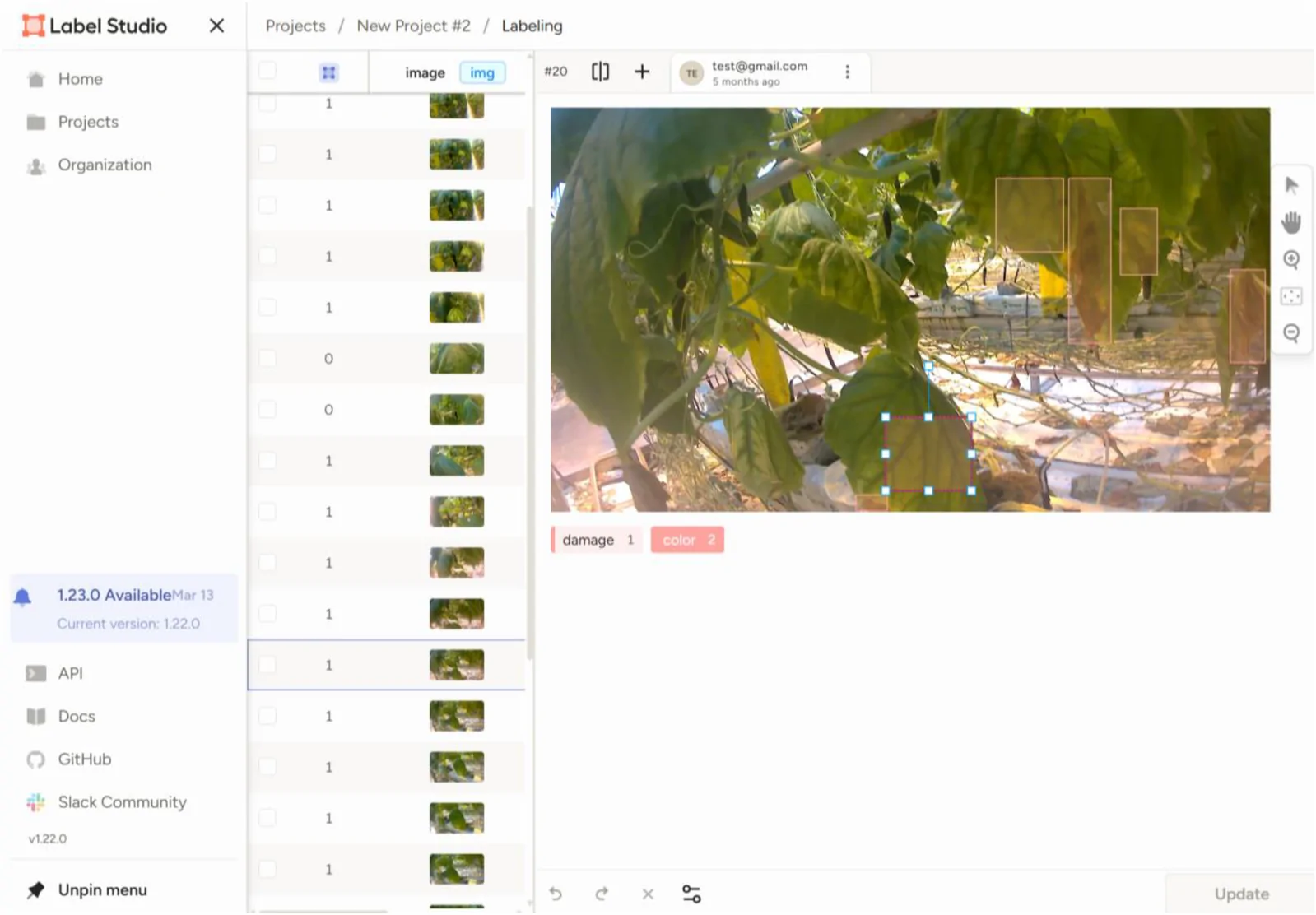
Rectangle annotation of visible cucumber leaf abnormalities in Label Studio. Multiple regions may be labeled within one image. Class 0 (color) denotes visible non-green discoloration, whereas class 1 (damage) denotes visibly deteriorated tissue regardless of confirmed cause. The boxes describe spatial extent rather than etiological diagnosis. Source: Screenshot of Label Studio (HumanSignal; https://labelstud.io/), licensed under the Apache License 2.0.

The deposited notes.json identifies Label Studio as the annotation tool but does not preserve per-image annotator identity or assignment. Version 1 contains no recoverable independently double-labeled subset, blinded re-annotation, adjudication record, annotator-specific export, or per-box reviewer decision. It is therefore impossible to determine whether two contributors labeled the same images independently or to calculate an inter-annotator agreement statistic retrospectively. The manuscript does not claim demonstrated between-annotator consistency. The complete structural audit verifies format and coordinate consistency but is not a substitute for independent biological or spatial re-annotation. A future quality-controlled version should predefine the phenotype rules, independently double-annotate a stratified subset, report class-specific detection agreement and box IoU/matching thresholds, and document adjudication. Reuse requiring clinical-style diagnostic certainty, severity grading, or pixel-accurate contours should add qualified expert review and release those derived annotations separately.

### 2.4 Performed construction workflow and data organization

The performed workflow comprises acquisition, eligibility screening, condition grouping, rectangle annotation, integrity auditing, and public release (**Figure 2**). It excludes polygon annotation, pixel-mask generation, train-validation-test splitting, model benchmarking, and sample predictions because these were not completed as components of version 1.

**Figure 2:**
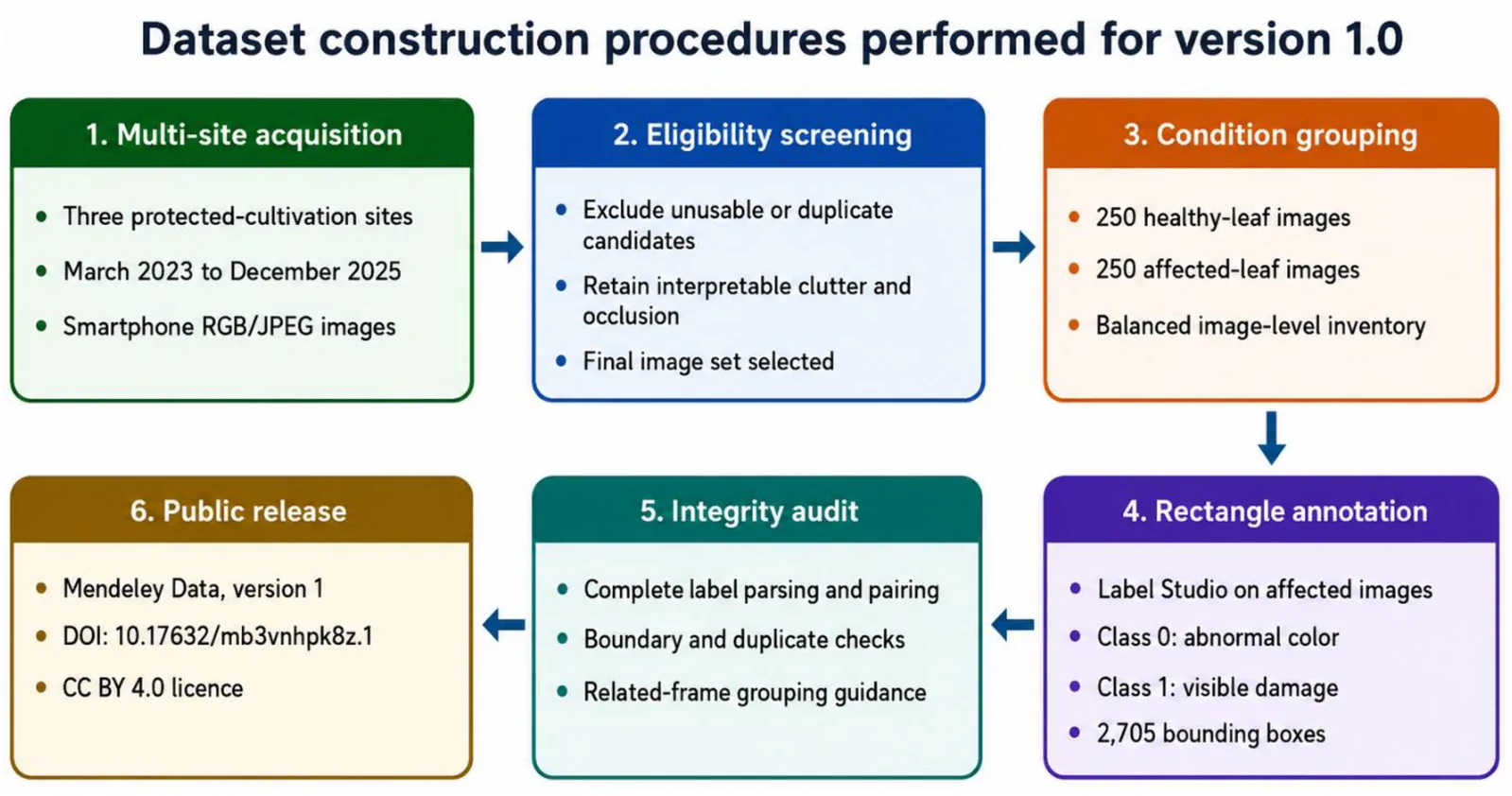
Procedures performed to construct, audit, and release dataset version 1.0. Only completed stages are shown.

The root directory contains Healthy leaves and Leaves with disease. These are immutable legacy directory names; Leaves with disease should be interpreted as the affected subset and not as pathogen confirmation. The deposited notes.json does not currently explain this terminology. A version 1.1 metadata patch should add an explicit note and user-facing affected-leaves alias while preserving version 1 and basename compatibility. The affected folder contains images, labels, classes.txt, and notes.json. Each affected image is paired with a TXT file having the same basename. A row contains class, x_center, y_center, width, and height, with the four spatial values normalized to image dimensions. Healthy images have no supplied boxes. No semantic or instance masks are included. **Supplementary Table S2** defines every directory, supplied file type, YOLO field, notes.json metadata element, and companion-manifest field group. Format conversions should preserve the original files and document every transformation (Lin et al., 2014).

### 2.5 Descriptive analysis and complete-release integrity checks

The public folder inventory, all 250 label files, and all 500 JPEGs were examined. Every JPEG decoded successfully, replacing the earlier metadata-only corruption screen. Every affected-image basename had a matching label basename. All label files were nonempty and all 2,705 rows contained five numeric tokens. Class identifiers were restricted to 0 and 1, and every center, width, and height was individually within the declared normalized range with positive dimensions. Color contributed 1,741 boxes (64.36%) and damage contributed 964 (35.64%), giving a mean of 10.82 boxes per affected image and a class-frequency ratio of 1.81:1. The image-level class-presence and overlap results are reported in Section 2.3 and **Table 2**.

**Table 2:** Dataset composition, quantitative audit findings, and required handling.

Item or check	Observed outcome	Interpretation or required action
Repository	Mendeley Data version 1; DOI 10.17632/mb3vnhpk8z.1; CC BY 4.0	Fixed public reference release; legacy title terms are explained
Image inventory	500 decoder-verified JPEGs: 250 healthy and 250 affected	Balanced curated groups; not a prevalence sample
Native dimensions	3840 × 2160 (387); 4608 × 3456 (112); 4000 × 2252 (1)	Modal/min 3840 × 2160; max 4608 × 3456
Filename information	113 date-bearing (22.6%); 387 sequence-derived (77.4%)	Coarse grouping only; not verified per-image acquisition metadata
Label inventory	250 nonempty TXT files; 2,705 rectangles; mean 10.82/affected image	One label file for every affected image
Class 0: color	1,741 boxes (64.36%); mean 6.96/affected image	Visible non-green color, not a causal diagnosis
Class 1: damage	964 boxes (35.64%); mean 3.86/affected image	Visible deterioration, including non-disease-associated damage
Image-level label presence	Both 215 (86.0%); color only 24 (9.6%); damage only 11 (4.4%)	Classes coexist and are not mutually exclusive
Cross-class spatial overlap	255 pairs in 116 images using IoU > 10^-6^; median IoU 0.0159; max 0.3391; 32 pairs ≥ 0.10; none ≥ 0.50	No class precedence; multi-target interpretation required
Box area	0.00395%–43.654% of image; median 0.800%; IQR 0.314%–1.813%; 182 boxes < 0.1%	Includes small-region localization challenges
Aspect ratio	Absolute ratio 1.00–32.00; median 2.49; IQR 1.61–3.84; p95 7.46	Includes elongated regions
Image-label pairing and syntax	250/250 basenames matched; all 2,705 rows had five numeric fields; 0 invalid class IDs	Preserve basename and five-field YOLO schema
Derived boundaries	42 boxes in 36 files; max overshoot 0.3278; sequence annotation rate 2.80% vs dated 0.90%	Correct, clip, or exclude; batch cause cannot be resolved from version 1
Exact duplicates	19 pairs/38 files: 13 affected pairs and 6 healthy pairs; 0 cross-folder pairs	Deduplicate or keep every pair within one split
Coarse perceptual candidates	3 non-exact high-similarity pairs/6 files; 2 within affected, 1 cross-folder	Group during splitting; manually review labels; screen is not exhaustive
Related frames	43 paired-name groups (86 files); 10 conservative sequence groups	Use group-aware splitting; do not randomly split adjacent frames
Unsupported labels	No pathogen diagnoses and no pixel-level masks	Do not report disease-species detection or segmentation ground truth

Normalized box area ranged from 0.00395% to 43.654% of image area (median 0.800%, mean 1.776%, interquartile range 0.314%–1.813%, and 95th percentile 6.798%). There were 182 boxes (6.73%) below 0.1% of image area and four (0.15%) above 25%. Absolute aspect ratio, defined as the larger of width/height and height/width, ranged from 1.00 to 32.00 (median 2.49; interquartile range 1.61–3.84; 95th percentile 7.46). These distributions indicate a meaningful small-region and elongated-region detection challenge.

Corner conversion showed that 42 boxes in 36 label files extend beyond at least one normalized image boundary; 26 are color boxes and 16 are damage boxes, and the largest overshoot is 0.3278. A filename-derived cluster check found 26 boundary boxes among 928 annotations in 138 sequence-derived affected images (2.80%; 24 images affected) and 16 among 1,777 annotations in 112 date-bearing affected images (0.90%; 12 images affected). This concentration is compatible with a batch or export effect, but annotator identity, device, Label Studio project state, and export-batch provenance are unavailable, so a specific tool or annotator cause cannot be assigned. The errors must be corrected or clipped under a documented rule before training. An exhaustive SHA-256/byte comparison identified 19 byte-identical pairs: 13 pairs within the affected folder and six within the healthy folder, with no exact pair spanning folders. Filename parsing identified 43 paired-name groups containing 86 files and 10 conservative source-sequence groups. A coarse full-release near-duplicate pass using 64-bit perceptual hash (pHash distance ≤ 4) together with 64-bit difference hash (dHash distance ≤ 8), excluding exact duplicates, flagged three additional high-similarity pairs comprising six files. Side-by-side review showed adjacent or closely related views; two pairs are within the affected subset and one spans healthy and affected folders. These candidates require group-aware splitting and label review, and the deliberately conservative screen should not be interpreted as exhaustive. **Table 2** consolidates the findings, and **Supplementary Figure S1** visualizes a released boundary-crossing box without clipping.

### 2.6 Interpretation and recommended reuse

The two folders can support healthy-versus-affected image classification, while the rectangles directly support two-class object detection and region localization. Healthy images should remain negative scenes when evaluating false positives. The color and damage labels may coexist and should not be treated as mutually exclusive disease species. Because no pixel masks are supplied, segmentation metrics must not be calculated against the rectangles as if they were expert contours. Any masks created by prompting, interpolation, or additional annotation constitute derived data and require independent validation.

The final 250/250 balance was created by curation from an unreported candidate pool; it is not an estimate of the frequency of healthy and affected leaves at the three facilities. Unknown rejection counts, possible condition-aware selection, and visual eligibility screening can alter both phenotype severity and image-quality distributions. Class priors, calibrated probabilities, prevalence estimates, and deployment thresholds derived directly from the folder balance would therefore be biased. External or prospectively sampled validation is required for prevalence-sensitive claims.

Random image-level splitting is inappropriate because exact duplicates and related frames can cross partitions. A reproducible conservative group identifier can be generated from filenames as follows: first remove the eight-character annotation-export prefix when present; then remove a terminal frame counter and terminal A/B view marker. Date-time names should be grouped by acquisition date, and frame families should be grouped by their source prefix. This rule produced 10 conservative sequence groups. A complementary pair identifier should remove only terminal A/B markers and merge frame42A/frame42B views, producing 43 paired-name groups. The three perceptual-hash candidate pairs must also remain group-locked; because one candidate spans healthy and affected folders, its label status should be reviewed before any classification benchmark. All members of an exact-duplicate, paired-name, sequence, or perceptual-candidate group must remain within one training, validation, or test partition. Users should publish their split manifest.

Before reuse, investigators should preserve an untouched copy of version 1, correct or clip the 42 boundary-crossing boxes under a declared rule, remove or group the 19 exact duplicate pairs, and group and manually review the three perceptual candidates. The pHash/dHash thresholds used here provide a coarse documented pass, not proof that all near-duplicates have been found; reuse pipelines should repeat the screen with task-appropriate transformations and visual confirmation. Dataset limitations should be reflected in the task definition: outputs indicate visible abnormal color or damage, not a specific pathogen or cause. Detailed model comparisons, new scientific findings, corrected labels, pixel masks, and enriched metadata should be published separately and linked to a new repository version where appropriate.

## Data Availability

The datasets presented in this study can be found in online repositories. The names of the repository/repositories and accession number(s) can be found below: https://data.mendeley.com/datasets/mb3vnhpk8z/1.

## Supplementary material

Supplementary Table 1 Operational inclusion and exclusion decision rules for release-level image selection.

Supplementary Table 2 Data dictionary for release directories, files, annotation fields, deposited metadata, and the companion manifest.

Supplementary Figure 1 Representative release images and annotations. **(A)** Example from Healthy leaves; the designation reflects visual screening and does not exclude latent abnormality. **(B)** Affected image containing class-0 color boxes (teal) and a class-1 damage box (orange), illustrating non-exclusive phenotype targets. **(C)** Released boundary-audit example rendered without clipping; the dashed purple class-0 rectangle extends below the image frame and has a normalized overshoot of 0.3278. Filenames are provided in the figure for reproducibility.
