# Supplementary material for: An annotated cucumber leaf image dataset for detection of visible damage and abnormal color symptoms in complex cultivation environments
Source: Front Plant Sci. 2026 Oct 2;17:1962647. doi: 10.3389/fpls.2026.1962647 (PMC13673411; doi:10.3389/fpls.2026.1962647)
Supplement: Supplementary file 1 [file SupplementaryFile1.docx]

# Supplementary Material

Supplementary Table S1. Operational inclusion and exclusion decision rules for release-level image selection.

| **Decision stage** | **Include or retain** | **Exclude** | **Recorded limitation** |
| --- | --- | --- | --- |
| **File readability** | JPEG decodes and can be visually inspected | Corrupted, unreadable, or failed decode | Original rejection count unavailable |
| **Target visibility** | At least one identifiable cucumber leaf is visible | No identifiable cucumber leaf | Plant identity was visual; plant IDs unavailable |
| **Focus and motion** | Leaf condition remains interpretable despite mild softness | Severe blur/motion prevents phenotype judgment | No numeric sharpness threshold was recorded |
| **Exposure** | Relevant leaf tissue remains interpretable despite highlight/shadow | Over- or underexposure makes condition uninterpretable | No calibrated illumination threshold was recorded |
| **Scene complexity** | Clutter, supports, overlap, scale variation, and partial occlusion retained | Only when complexity removes an interpretable leaf region | Complex scenes were intentionally preserved |
| **Initial duplicate screen** | Visually distinct or not recognized as duplicate at initial selection | Obvious duplicate detected during initial manual selection | Later audit still found 19 exact pairs |
| **Healthy assignment** | No clear visible non-green departure or structural damage on visible cucumber leaves | Clear visible abnormality; move to affected if interpretable | Visual judgment only; subtle/latent abnormalities not excluded biologically |
| **Affected assignment** | At least one clear visible color departure or tissue-damage phenotype | No visible phenotype, or phenotype uninterpretable | No etiological or pathogen confirmation |
| **Ambiguous but interpretable cause** | Retain and label the observable phenotype without causal diagnosis | Do not assign a pathogen/disease subtype | Color and damage may coexist |
| **Final balancing** | Manual selection continued until 250 healthy and 250 affected images were retained | Additional otherwise eligible images were not documented | Not a prevalence sample; initial pool and attrition unknown |

Supplementary Table S2. Data dictionary for release directories, files, annotation fields, deposited metadata, and the companion manifest.

| **Element** | **Type/format** | **Meaning** | **Availability or constraint** |
| --- | --- | --- | --- |
| **Dataset/** | Root directory | Top-level fixed release | Contains two legacy subset directories |
| **Healthy leaves/** | Directory | 250 visually healthy JPEG images | No supplied label files; ‘healthy’ is visual, not biological confirmation |
| **Leaves with disease/** | Directory | Legacy name for the 250-image affected subset | Name does not establish disease; version 1 immutable |
| **Leaves with disease/images/** | Directory | 250 affected JPEG images | Each basename maps to one TXT file |
| **Leaves with disease/labels/** | Directory | 250 YOLO-format TXT files | One nonempty file per affected image |
| **classes.txt** | UTF-8 text | Ordered class names: color; damage | Class IDs 0 and 1 |
| **notes.json** | JSON | Label Studio export metadata | No per-image site/device/annotator provenance |
| **notes.json categories[*].id/name** | Integer/string | 0/color and 1/damage label mapping | Deposited values |
| **notes.json info.year** | Integer | Dataset/export year (2026) | Release-level only |
| **notes.json info.version** | String | Internal value 1.0 | Distinct from Mendeley version label 1 |
| **notes.json info.contributor** | String | Label Studio | Tool/export contributor, not an annotator identity |
| ***.jpg** | RGB JPEG | Source image | Dimensions: 3840 × 2160, 4608 × 3456, or 4000 × 2252 |
| **TXT field 1: class** | Integer | 0 = color; 1 = damage | Phenotype, not causal diagnosis |
| **TXT field 2: x_center** | Normalized float | Horizontal box center/image width | Declared 0–1; corner conversion can exceed frame |
| **TXT field 3: y_center** | Normalized float | Vertical box center/image height | Declared 0–1; corner conversion can exceed frame |
| **TXT field 4: width** | Normalized positive float | Box width/image width | Positive; individual value ≤ 1 |
| **TXT field 5: height** | Normalized positive float | Box height/image height | Positive; individual value ≤ 1 |
| **Manifest identity fields** | CSV strings | filename; relative_path; subset_class | One row per image (500 rows) |
| **Manifest acquisition fields** | CSV strings | site; device; filename_category; acquisition_date; acquisition_time_from_filename; source_session_group | site/device = NA; date/time/group derived from filenames where possible |
| **Manifest image fields** | CSV numeric/string | width_px; height_px; decoder_status; file_size_bytes | Computed from fixed release |
| **Manifest annotation fields** | CSV numeric/string | label_filename; annotation_count_total; annotation_count_color; annotation_count_damage; cross_class_overlap_pairs; boundary_crossing_box_count | Healthy rows use NA/zero as applicable |
| **Manifest integrity fields** | CSV string | exact_duplicate_group; perceptual_candidate_group; sha256; phash64; dhash64 | Group IDs are audit-derived; NA means not flagged |
| **Unavailable per-image metadata** | NA | Exact site, device, plant, cultivar, distance, illumination, annotator, verified acquisition session | Not recoverable from version 1 and not inferred |

# Supplementary Figure S1

Supplementary Figure S1. Representative release images and annotations. (A) Example from Healthy leaves; the designation reflects visual screening and does not exclude latent abnormality. (B) Affected image containing class-0 color boxes (teal) and a class-1 damage box (orange), illustrating non-exclusive phenotype targets. (C) Released boundary-audit example rendered without clipping; the dashed purple class-0 rectangle extends below the image frame and has a normalized overshoot of 0.3278. Filenames are provided in the figure for reproducibility.
